# Prevalence of communicable, non-communicable diseases, disabilities and related risk factors in Khyber Pakhtunkhwa Pakistan: Findings from the Khyber Pakhtunkhwa Integrated Population and Health Survey (2016–17)

**DOI:** 10.1371/journal.pone.0308209

**Published:** 2025-02-03

**Authors:** Ziaul Haq, Saima Afaq, Muhammad Ibrahim, Zala, Muhammad Asim

**Affiliations:** 1 Institute of Public Health & Social Sciences (IPH&SS), Khyber Medical University (KMU), Peshawar, Pakistan; 2 Institute of Health & Wellbeing, University of Glasgow, Glasgow, United Kingdom; 3 Department of Epidemiology and Biostatistics, School of Public Health, Imperial College, London, United Kingdom; 4 Department of Health Sciences, University of York, Heslington, York, United Kingdom; Aga Khan University, PAKISTAN

## Abstract

**Introduction:**

Pakistan is facing a triple burden of diseases: communicable diseases (CDs), non-communicable diseases (NCDs) and disabilities. There is limited evidence on the patterns of these diseases in Pakistan, specifically Khyber Pakhtunkhwa. Additionally there remains limited study on the impact of sex-, age and setting-stratified morbidities in Khyber Pakhtunkhwa.

**Objectives:**

The objective of this study was to present the demographic characteristics and the burden of locally-specific CDs (hepatitis B and C, TB, AIDS), NCDs (diabetes, renal diseases, asthma, epilepsy, coronary heart diseases, cancer, hypertension, cholesterol, thalassemia), and disabilities (congenital, post-disease, post-injury, paralysis) stratified by sex, age and setting in the Khyber Pakhtunkhwa province of Pakistan.

**Material and methods:**

The Khyber Pakhtunkhwa Integrated Population and Health Survey (KP-IPHS) was conducted in 2016–17 to gather comprehensive information about the demographic characteristics and locale-specific health-related issues of the people of Khyber Pakhtunkhwa, Pakistan. This cross-sectional survey was conducted in 24 districts across all 7 divisions of Khyber Pakhtunkhwa on population ageing ≥18 years. A total of 20704 respondents were taken from primary (n = 1061) and secondary sampling units (n = 15724), designed with considerations for urban/rural and socio-economic status. Each primary unit included 250–300 households. The sample selection utilised a multi-staged stratified systematic cluster sampling technique, involving the inclusion of every 16th household in rural areas and every 12th household in urban areas. Observations were recorded on demographics, quality of life, physical activity, BMI, disabilities, CDs and NCDs.

**Results:**

Among all NCDs, hypertension has the highest prevalence (29.2%), showing a significant difference between females (32.7%) and males (25.0%). The proportion of males and females with diabetes is 7.4% and 5.2%, respectively, with a more substantial percentage (11.2%) observed in the ≥50 years’ age group compared to 2.5% in the 18–29 years’ age group and 5.0% in the 30–49 years’ age group. Among the included CDs, hepatitis B and C (0.5% and 0.8%, respectively) are most prevalent. Hepatitis B is more common in females (0.8%) and in rural settings (0.6%) compared to males (0.3%) and urban settings (0.2%), respectively. The prevalence of congenital disabilities is the highest of all disabilities, with a significantly high prevalence in females (5.3%) and rural settings (3.5%) compared to males (0.9%) and urban settings (0.6%) respectively. Furthermore, post-injury, post-disease disabilities, and paralysis significantly increase with age (P < 0.001).

**Conclusions:**

Our findings show that the prevalence of CDs, NCDs and disabilities varies across sex, age and settings, with a notable prevalence in females and the old-age population (≥50 years). The results emphasise the necessity of public health efforts, such as screening, prevention, and treatment, to tackle the triple burden of CDs, NCDs, and disabilities in Khyber Pakhtunkhwa Pakistan.

## Introduction

The double burden of communicable diseases (CDs) and non-communicable diseases (NCDs) is becoming increasingly prevalent worldwide, affecting both developed and developing countries [1]. However, developing countries are particularly vulnerable due to a range of factors such as geographic, demographic, and socioeconomic disparities [2]. The burden of CDs and NCDs is significantly associated with economic and social development worldwide, hindering progress, posing threats to international health security, and imposing significant financial burdens on health systems [3–7].

CDs have always been a major public health concern in LMICs [8, 9]. In 2019, CDs accounted for 18% of global deaths, with Pakistan contributing approximately 39% of these deaths. Diarrheal diseases, lower respiratory infections, tuberculosis (TB), malaria, and human immunodeficiency (HIV)/AIDS remain in the top-10 causes of death globally [10–12]. Worldwide, Pakistan is the fourth highest country in prevalence of multi-drug resistant tuberculosis (MDR-TB) [13]. In Pakistan, an estimated 11% of adult population is infected with the hepatitis C virus (HCV), making it the second highest country in the prevalence of HCV globally [14], which increases the risk of chronic liver disease and cancer [14]. HIV/AIDS prevalence in the general population in Pakistan remains below 1% [15].

Lately, there has been a shift in the global disease burden to NCDs which, according to WHO, has been caused by the decline in premature mortality from CDs, particularly in low-income countries (LICs) and low- and middle-income countries (LMICs) including Pakistan [3, 6, 16, 17]. Every year, NCDs kill 41 million people, which is equivalent to 74% of all fatalities worldwide [6]. More than 17 million people die each year from an NCD worldwide before age 70, with LMICs accounting for 86% of these premature deaths [6]. Cardiovascular diseases account for the majority of NCD mortality, accounting for 17.9 million deaths per year, followed by cancers (9.3 million), chronic respiratory diseases (4.1 million), and diabetes (2.0 million) [6]. The two main risk factors for deaths from cardiovascular diseases, cancers, and respiratory diseases are tobacco use and high blood pressure [18, 19]. In Pakistan, NCDs are among the top causes of death among which diabetes, cancer, hypertension, mental health disorders, accidents, and injuries accounted for 55% deaths in 2019 [10, 18].

In addition to the CDs and NCDs, disabilities are an emerging global issue, affecting nearly 1 billion people worldwide, representing approximately 15% of the global population [20]. The prevalence of disabilities is increasing due to a rise in NCDs and an extended average lifespan within the population. Individuals with disabilities face a twofold higher risk of developing conditions like depression, asthma, diabetes, stroke, or obesity [21–23]. In Pakistan, an estimated 2.5% of the population was reported to be disabled based on the 1998 census, while according to the 2017 census the disability burden is approximately 0.5% [24–27]. According to WHO, 2–4% of those with disabilities experience significant difficulties in functioning and this proportion is even higher in developing countries [26, 28]. In Pakistan, disabilities due to congenital factors, including neurological disorders and limb defects, as well as post-disease and post-injuries are recognised as significant contributors to lifelong disabilities in the country [29–32].

Nonetheless, Pakistan, a disaster-prone, impoverished, and terrorism-stricken country, is facing a triple burden of health issues: CDs, NCDs and disabilities [33–35]. The government of Pakistan spends only 2.95% of its total GDP on healthcare, considerably lower than the LMICs (5.62%) and the global average (10.89%), limiting access to affordable healthcare for impoverished populations [36–38]. Despite significant progress, the targets of Sustainable Development Goal SDG-3 "ensuring health and well-being for all by 2030" are unlikely to be met by 2030 due to rising triple burden of these health issues in the country.

Khyber Pakhtunkhwa is a province located in the northwestern region of Pakistan. It is the fourth-largest province in Pakistan in terms of land area and the third-largest in terms of population. The province covers an area of 101,741 square kilometers and has a population of approximately 35,519,927 people [39, 40]. The earlier health surveys conducted in Khyber Pakhtunkhwa, including the Pakistan Demographic and Health Survey (PDHS) 2017–2018 [41] and the Khyber Pakhtunkhwa Multiple Indicator Cluster Survey (MICS) 2019 [42], do not adequately cover all health concerns across every region of the Khyber Pakhtunkhwa province. For instance, the PDHS 2017–2018 is representative for four provinces and other regions of Pakistan, but its representation for all districts within Khyber Pakhtunkhwa is unclear. The KP-MICS 2019 provides estimates for the seven divisions and 25 districts of the Khyber Pakhtunkhwa, yet may not cover all aspects of health in the province, leaving gaps in available data. These gaps limit the understanding of the health situation in Khyber Pakhtunkhwa and the development of effective policies and programs. We conducted the Khyber Pakhtunkhwa Integrated Population and Health Survey (KP-IPHS), which aims to fill these gaps by providing comprehensive data on various health indicators, including the leading CDs, NCDs, and disabilities in the province; and to influence local health policy frameworks in the country. It also aims to provide valuable information for the global dialogue on healthcare disparities in Pakistan and to guide international health agendas by advocating for strategies that promote universal health coverage and equitable healthcare access and delivery in LMICs including Pakistan.

## Materials and methods

The Khyber Pakhtunkhwa Integrated Population and Health Survey (KP-IPHS) [43] was conducted in 2016–17 to gather comprehensive information about the demographic characteristics and locale-specific health-related issues of the people of Khyber Pakhtunkhwa, Pakistan. This cross-sectional survey was conducted in 24 districts of Khyber Pakhtunkhwa, ensuring a balanced representation of all 7 divisions of Khyber Pakhtunkhwa and the diverse geographical and demographic characteristics of the province.

### Objectives

The objective of this study was to present the demographic characteristics and the burden of locally-specific CDs (hepatitis B and C, TB, AIDS), NCDs (diabetes, renal diseases, asthma, epilepsy, coronary heart diseases, cancer, hypertension, cholesterol, thalassemia), and disabilities (congenital, post-disease, post-injury, paralysis) stratified by sex, age and setting in the Khyber Pakhtunkhwa province of Pakistan.

### Eligibility criteria

Our study included individuals aged 18 years or older residing in both rural and urban areas of the Khyber Pakhtunkhwa province, Pakistan. Participation in the study was voluntary, and individuals willing to partake in the survey were eligible. Individuals unable to provide written informed consent, not present during the survey period, or falling outside the adult age range were excluded.

### Data collection tools

A comprehensive Health Questionnaire was developed to gather information on demographics, body mass index (BMI), quality of life (QOL), physical activity (PA), and data on various health conditions, including CDs, NCDs, and disabilities. The participants were asked to self-report whether they had ever been diagnosed with any of these conditions by a health professional. The questionnaire was developed by integrating other standard model questionnaires, which were customised to meet the needs and conditions of the province in the Urdu language, including the International Physical Activity Questionnaire (IPAQ), questions on anthropometry, and variables related to CDs, NCDs and disabilities, and the World Health Organisation (WHO) Quality of Life Questionnaire (WHOQOL-BREF).

Weight and height were measured following standardised protocols by the trained field workers using portable equipment. Weight measurements, taken without shoes or head turbans, were recorded to the nearest 0.1 kg using digital scales on a hard and flat surface. Height measurements were recorded to the nearest 0.1 cm using a stadiometer on a hard and flat surface. Three measurements were taken, and an average of these values was documented.

The IPAQ is a 27-item self-report questionnaire that measures the duration and intensity of different types of physical activities undertaken by an individual during a week, encompassing work-related, leisure, and household tasks [44].

The WHOQOL-BREF is a 26-item self-administered questionnaire designed to assess the subjective evaluation of an individual’s perceived quality of life over the previous two weeks in four domains: physical health, social relationships, psychological health, and environment [45].

### Sample size and sampling strategy

The Bureau of Statistics at the Planning and Development Department, Khyber Pakhtunkhwa (http://kpbos.gov.pk/) [46] assisted in determining the sample size and selecting sampling points, maps, and household listings. To recruit adult males and females over the age of 18, the study employed a multistage stratified cluster sampling technique. The Pakistan Bureau of Statistics (PBS) conducted sampling in the 24 districts, based on urban and rural areas, creating a unique sampling frame using the updated list of enumeration blocks for urban areas from the Economic Census of 2003 and the list of villages/mohallas/dehs for rural areas from the 1998 Population Census. Line listing of all these selected enumeration blocks for urban and mohallas/villages for rural areas was done by the field team to update the sampling frame and household number. In the context of the urban sampling frame, each city or town was divided into several enumeration blocks. These blocks typically comprised 250–300 households as per the updated line listing and were characterised by clear boundaries and maps. According to the PBS, large-sized cities or districts with a population of 500,000 or more were considered independent urban strata throughout Pakistan. Consequently, in the province of Khyber Pakhtunkhwa, only the Peshawar district was classified as such. Each of these cities was then further subdivided by PBS into low, middle, and high-income groups based on the living standards of the majority of households and the information collected from each enumeration block. The rest of the cities/towns within each defunct administrative division were grouped to form an independent urban stratum. The entire rural area of each district is considered an independent rural stratum.

The sampling frame for urban areas consists of 1913 enumeration blocks that have been updated from the Economic Census of 2003. On the other hand, the sampling frame for rural areas consists of 7337 villages based on the 1998 census. In this framework, enumeration blocks and villages/mohallas/dehs serve as Primary Sampling Units (PSUs) for urban and rural domains, respectively. To select sample PSUs from each stratum/sub-stratum in urban areas, the probability proportional to size (PPS) method of sampling scheme has been used, with households in each block being the measure of size (MOS). Similarly, the population of each village has been taken as MOS for the selection of sample villages using the probability proportional to size method of selection in rural areas. The constituent households of sample PSUs were taken as Secondary Sampling Units (SSUs). With a random start, a specified number of households (12 from each urban sample PSU and 16 from rural sample PSU) have been selected with equal probability using systematic sampling technique. The total number of households/SSUs selected for data collection was 15,724 from 1061 PSUs, comprising 3,756 SSUs from 313 urban PSUs and 11,968 SSUs from 748 rural PSUs selected at random [47, 48]. The sample size was designed to detect estimates with a 95% confidence interval and a 5% margin of error.

Within each SSU (household), one eligible adult male and one eligible adult female aged 18 or older who were present and provided consent were included in the study. In cases where a household refused participation or was found unoccupied after two repeated visits by the field team, the household was classified as non-response and no replacement was made. These non-responding households were accounted for during the calculation of sample weights to maintain the representativeness of the final results.

### Data collection and quality assurance

Trained field workers conducted interviews with individual household members, ensuring accurate and detailed responses. The data collection process involved approximately 30–40 field workers from the Population Welfare Department of Khyber Pakhtunkhwa per district, with monitoring teams consisting of demographers (Public Works Department—PWD Officers) from each of the 24 districts, who conducted random visits to the data collection points. To equip demographers with the necessary skills, a comprehensive two-day orientation and feedback training session on survey tools, including sample selection and household line listing procedures, was conducted. This training was facilitated by a consultant from the Bureau of Statistics, bringing expertise gained from involvement in the Multiple Indicator Cluster Survey (MICS) of 2007 and 2016. Moreover, the district-level training of field workers, led by a trained team comprising faculty and researchers from KMU under the Primary Investigator, focused on ensuring a thorough understanding of the tools and overall data collection process.

### Data handling and analysis

A special database was designed in the Microsoft Epi Info for data entry. All data was transferred from the hard forms to the database during the active data collection phase and then was crosschecked with the hard forms, to get the most authentic data for analysis. The cleaned data was transferred into SPSS version 29 for analysis. Hard copies of the tools were stored with proper cataloguing and the soft data was saved in the Khyber Medical University official system following the data security and handling SOPs.

Metabolic equivalents (METs), defined as the amount of oxygen required during rest calculated per one kilogram of body weight and time [49], for physical activity were calculated by using the guidance provided by the International Physical Activity Questionnaire IPAQ [50]. MET values for low (including walking), moderate and vigorous intensity activities, were set at 3.3, 4 and 8 respectively. Subsequently, the MET minutes per week for each activity category were calculated by multiplying the duration in minutes, frequency in days per week, and the respective MET value. The total MET minutes per week was obtained by summing the MET minutes for walking, moderate-intensity, and vigorous-intensity activities. The interpretation of activity levels was categorised as low, moderate, or high based on the total MET minutes per week—less than 600 for low activity, between 600 and 1500 for moderate activity, and greater than 1500 for high activity [51].

For quality of life, the raw scores from the WHOQOL-BREF questionnaire’s four domains: physical health, social relationships, psychological health, and environmental health were transformed to a scale of 0 to 100 using the formula below [52].


$$Transformed\;scale=\left[\frac{\left(actual\;raw\;score-minimum\;possible\;raw\;score\right)}{possible\;range\;of\;raw\;score}\right]*100$$

Body Mass Index (BMI) was derived by dividing the weight of an individual in kilograms by the square of their height in metres. The survey participants were categorised based on their BMI values according to the World Health Organisation guidelines: underweight (BMI < 18.5), normal weight (BMI 18.5–24.9), pre-obesity (BMI 25.0–29.9), obesity class I (BMI 30.0–34.9), obesity class II (BMI 35.0–39.9), and obesity class III (BMI above 40) [53].

Descriptive statistics, including frequency percentages for categorical variables, and means with standard deviation (SD) for scale variables, are presented. The independent t-test and One-way ANOVA were used to compare means between two and more than two independent variables, respectively. Stratification of the prevalence of CDs, NCDs and disabilities across various demographic characteristics such as sex, age, and setting was conducted. Pearson’s Chi-squared test was employed to evaluate significant differences in prevalence between groups. A P-value of 0.05 or less was considered statistically significant, whereas a P-value of 0.05 or more was considered to indicate no association between these. For reporting all results from the survey data, sample weights are used.

### Handling missing data

Detailed information regarding the missing data is available in Appendix A in S1 Text. On average, for 88% of the variables, less than 30% of the data was missing. To deal with the missing data, we employed pairwise deletion, a method that is well-suited to Missing Completely at Random (MCAR) or Missing at Random (MAR) data [54]. Given the variation in missing data across variables in our study, pairwise deletion was deemed advantageous as it is less biased under MCAR or MAR assumptions and preserves more information than other deletion methods.

### Ethical considerations

The survey received ethical approval from the Research Ethical Committee of Khyber Medical University. Written informed consent was taken from all the participants. For participants who were unable to read, the interviewer read the consent form to them and received their consent. All participants were assured of their right to withdraw from the study at any time without any consequences. Data confidentiality was ensured by restricting data accessibility to the Primary Investigator only.

## Results

**Fig 1:**
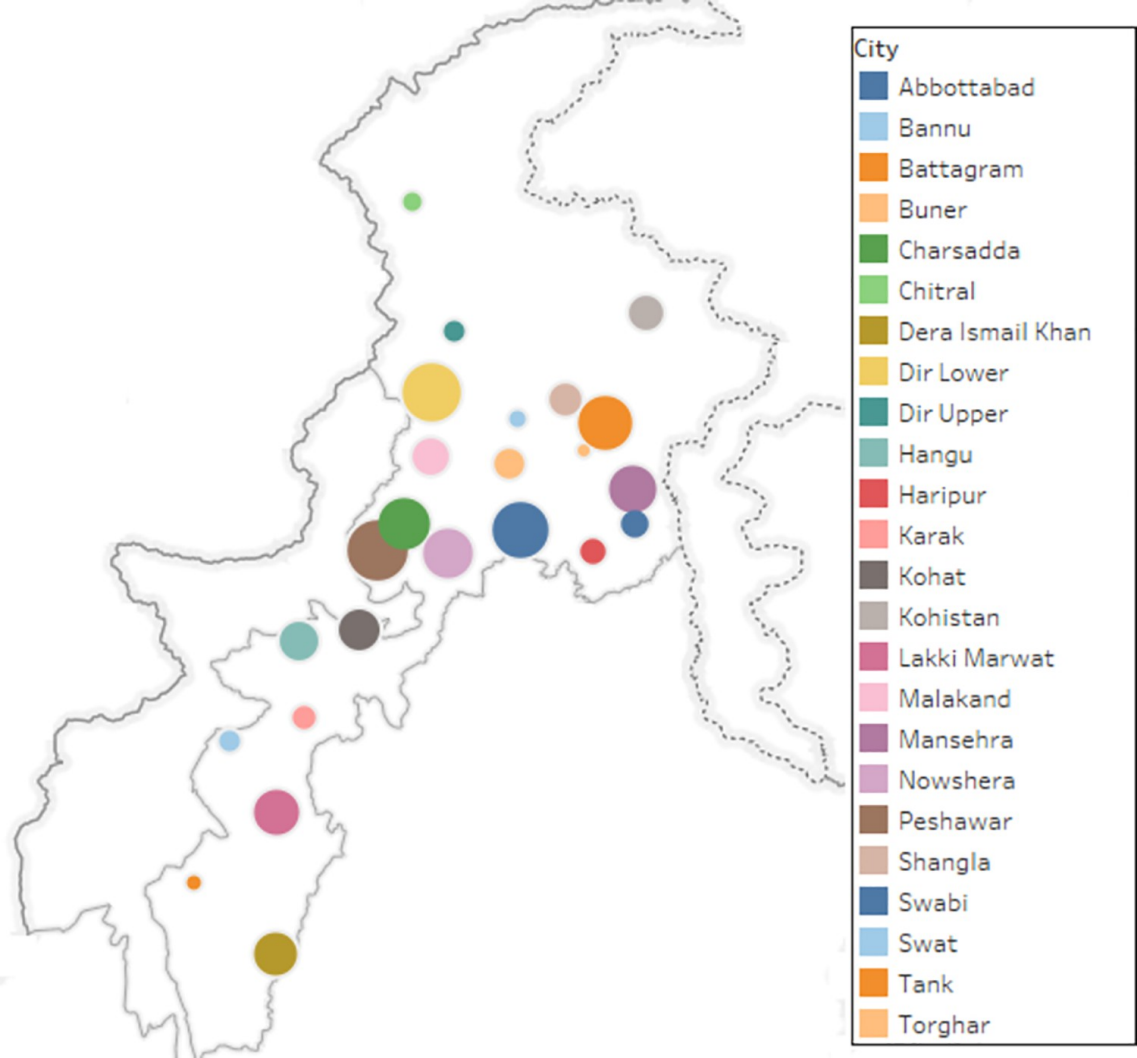
Sample size mapping of the 24 districts in Khyber Pakhtunkhwa.

(Fig 1) illustrates the sample size mapping of the 24 districts in Khyber Pakhtunkhwa. The colour and size of circles vary with districts’ location and sample size, respectively. This comprehensive dataset comprises responses from 20,704 individuals gathered from 15,724 SSUs/households, with an overall response rate of 71.5%. Maximum respondents (n = 1324) were from district Peshawar, followed by Dir Lower (n = 1277) and Swabi (n = 1207). Torghar district recorded the lowest number of respondents (n = 153).

### Demographic characteristics of study participants

Table 1 presents a comprehensive overview of the sociodemographic characteristics of the study participants. The proportion of males and females was almost similar (48.60% vs. 51.40%, respectively). Age-wise, the majority of participants were within the 30–49 age group (56.50%), followed by those aged 50 and above (26.80%), and then those aged 18–29 (16.70%). The educational background of participants varied, with a high percentage having completed primary school (33.20%). The proportion of respondents who completed secondary school was 32.60%, while those who finished college (12 years) education made up 13.80%. Most participants were from rural settings, representing 87.00% of the sample, while 13.00% were from urban areas. In terms of occupation, the study includes participants with diverse employment statuses, including government employees (9.10%), non-government employees (8.90%), and self-employed individuals (23.00%). However, a high proportion (59.00%) of respondents were unpaid.

**Table 1:** Sociodemographic characteristics of participants.

Characteristics	N	Percentage (%)
**Sex**		
Male	9208	48.60%
Female	9742	51.40%
**Age groups (years)**		
18–29	3166	16.70%
30–49	10683	56.50%
>50	5067	26.80%
**Setting**		
Rural	16587	87.00%
Urban	2480	13.00%
**Education**		
No formal schooling	531	6.90%
Some primary schooling	490	6.40%
Primary school completed	2550	33.20%
Secondary school completed	2507	32.60%
College (12 years) completed	1062	13.80%
Bachelors completed	480	6.20%
Postgraduate degree	63	0.80%
**Occupation**		
Government employee	1617	9.10%
Non-government employee	1575	8.90%
Self-employed	4091	23.00%
Unpaid*	10491	59.00%

*Students, housewives, retirees and unemployed people.

### Body mass index (BMI)

Table 2 provides Body Mass Index (BMI) data stratified by sex, age, and setting. When stratified by sex, the mean BMI was significantly different between males and females (P < 0.001), with females having a higher mean BMI of 25.8 (SD = 7.0) compared to males, 23.7 (SD = 4.6). The prevalence of underweight, pre-obesity, and obesity (Class I, II, III) was higher among females, constituting a total of 10.2% in the underweight and 50.4% in the pre-obesity or obesity categories among all female participants, compared to 8.9% underweight and 33.3% in males.

**Table 2:** Body mass index-BMI (kg/m2) stratified by sex, age, and setting.

**BMI by Sex**	**All participants**	**Male**	**Female**	**P-value**
BMI Mean (SD)	24.75(6.0)	23.71(4.6)	25.85(7.0)	<0.001
Underweight	1225 (9.6%)	584(8.9%)	638(10.2%)	0.018
Normal Weight	6258(48.8%)	3769(57.8%)	2471(39.4%)	<0.001
Pre-Obesity	3250(25.4%)	1581(24.3%)	1663(26.5%)	0.003
Obesity Class I	1392(10.9%)	465(7.1%)	924(14.8%)	<0.001
Obesity Class II	361(2.8%)	77(1.2%)	283(4.5%)	<0.001
Obesity Class III	331(2.6%)	45(0.7%)	286(4.6%)	<0.001
**BMI by Age group**	**18–29**	**30–49**	**≥50**	**P-value**
Mean BMI (SD)	24.32(6.1)	25.00(6.1)	24.71(6.04)	<0.001
Underweight	262 (12.1%)	649 (9.1%)	304 (8.9%)	<0.001
Normal Weight	1086(50.2%)	3401(47.7%)	1708 (49.9%)	0.03
Pre-Obesity	503(23.3%)	1863(26.1%)	872 (25.5%)	0.02
Obesity Class I	180(8.3%)	834(11.7%)	336 (10.7%)	<0.001
Obesity Class II	73(3.4%)	195(2.7%)	92 (2.7%)	0.24
Obesity Class III	56(2.6%)	189(2.6%)	83 (2.4%)	0.79
**BMI by Setting**	**Rural**	**Urban**	-	**P-value**
Mean BMI (SD)	24.70 (6.1)	25.09 (6.0)	-	<0.001
Underweight	1104 (9.7%)	121 (8.1%)	-	0.04
Normal Weight	5563 (49.1%)	695 (46.8%)	-	0.09
Pre-Obesity	2880 (25.4%)	370 (24.9%)	-	0.66
Obesity Class I	1187 (10.5%)	205 (13.8%)	-	<0.001
Obesity Class II	303 (2.7%)	58 (3.9%)	-	0.007
Obesity Class III	295 (2.6%)	36 (2.4%)	-	0.68

Case-wise total, valid & missing cases are given in Appendix Table A in S1 Text. BMI (kg/m2) is presented as Mean (±SD) and the remaining variables are presented as n(%).

The mean BMI significantly varied across different age categories (P < 0.001), with participants in the 30–49 age group having the highest mean BMI, 25.0 (SD = 6.1), followed by the ≥50 age group, 24.7 (SD = 6.0), and the 18–29 age group with the lowest mean BMI, 24.3 (SD = 6.1). Additionally, there was a significantly higher prevalence of underweight individuals in the 18–29 age group (12.1%) compared to the other age groups (P = 0.02). Similarly, pre-obesity and obesity Class I was highest among the 30–49 years age group (P < 0.001).

Significant differences were observed in mean BMI (P < 0.001), with urban areas showing a higher mean BMI of 25.09 (SD = 6.0) compared to rural areas, which had a mean BMI of 24.70 (SD = 6.1). Notably, the prevalence of underweight was significantly higher in rural areas (9.7%) compared to urban areas (8.1%), while obesity (Class I, II) was more prevalent in urban settings.

### Physical activity (MET-minutes)

Table 3 presents physical activity by metabolic equivalents in minutes (MET-minutes), stratified by sex, age, and setting. Total PA (MET-minutes) was significantly higher in males (3428.5) compared to females (1811.6) (P < 0.001). Likewise, males reported significantly higher vigorous PA (60.9%) compared to females (43.3%) (P < 0.001). Moderate PA showed no statistically significant difference between males and females (P = 0.07), while low PA displayed a significant difference (P < 0.001), with higher prevalence among females (32.9%).

**Table 3:** Physical activity (MET-minutes) stratified by sex, age, and setting.

**Physical Activity (MET-minutes) by Sex**	**All Participants**	**Male**	**Female**	**P-value**
Total Physical Activity (METs-minutes)	2731.9 (3861.7)	3428.5 (4610.5)	1811.6 (2254.7)	<0.001
Vigorous PA	4444 (53.9%)	2893 (60.9%)	1541 (43.3%)	<0.001
Moderate PA	1884(22.8%)	1034 (22.1%)	846 (23.8%)	0.07
Low PA	1918 (23.3%)	745 (15.9%)	1172 (32.9%)	<0.001
**Physical Activity (MET-minutes) by Age Group**	**18–29**	**30–49**	**≥50**	**P-value**
Total Physical Activity (METs-minutes)	2708.6 (3775.9)	2654.9 (3651.8)	2946.0 (4389.2)	0.01
Vigorous PA	699 (52.9%)	2586(54.2%)	1121 (53.9%)	0.71
Moderate PA	252 (19.1%)	1096 (23.0%)	520 (25.0%)	<0.001
Low PA	370 (28.0%)	1091 (22.9%)	438 (21.1%)	<0.001
**Physical Activity (MET-minutes) by Setting**	**Rural**	**Urban**	**-**	**P-value**
Total Physical Activity (METs-minutes)	2746.4 (3868.9)	2634.3 (3813.7)	-	0.37
Vigorous PA	3919 (54.6%)	525 (49.4%)	-	0.001
Moderate PA	1593 (22.2%)	291 (27.4%)	-	<0.001
Low PA	1670 (23.3%)	248 (23.3%)	-	0.97

Case wise total, valid & missing cases are given in Appendix Table A in S1 Text. Total Physical Activity (MET-minutes) is presented as Mean (±SD), and the remaining variables are presented as n(%).

A significant difference was observed in total PA levels across the age groups 18–29, 30–49, and ≥50 (P = 0.01). While no statistically significant difference was found in the prevalence of vigorous PA among different age groups (P = 0.7), notable variations were observed in moderate and low PA. Moderate PA demonstrated a significant increase with age (P < 0.001), with the highest prevalence in the ≥50 age group (25.0%). In contrast, low PA exhibited a significant decrease with age (P < 0.001), with the lowest prevalence observed in the ≥50 age group (21.1%).

Total PA (MET-minutes) did not demonstrate a significant difference between rural (2746.4) and urban (2634.3) settings (P = 0.37). Low PA did not reveal a statistically significant difference between rural and urban settings (P = 0.97. At the same time, vigorous and moderate PA showed significant differences (P = 0.001, P < 0.001), with a higher prevalence of vigorous PA in rural areas (54.6%) and moderate PA in urban areas (27.4%).

### Quality of life (QOL)

Table 4 summarises results for all four domains of quality of life on the WHOQOL-BREF questionnaire, stratified by sex, age, and setting. Mean scores for the physical, psychological, social relations and environmental domains were 54.4 (11.3), 53.7 (13.2), 64.5 (21.2), and 52.3 (15.7), respectively. Significant differences were found across all domains.

**Table 4:** Quality of life (QOL) stratified by sex, age, and setting.

**QOL by Sex**	**All Participants Mean (SD)**	**Male Mean (SD)**	**Female Mean (SD)**	**P-value**
Physical	54.39 (11.3)	54.96 (10.7)	53.83 (11.9)	<0.001
Psychological	53.72 (13.2)	54.75 (13.1)	52.79 (13.13)	<0.001
Social	64.53 (21.2)	66.83 (20.1)	62.38 (22.0)	<0.001
Environmental	52.29 (15.7)	53.08 (15.3)	51.58 (16.1)	<0.001
**QOL by Age Group**	**18–29 Mean (SD)**	**30–49 Mean (SD)**	**≥50 Mean (SD)**	**P-value**
Physical	54.63 (11.8)	54.61 (11.2)	53.57 (11.3)	0.001
Psychological	54.40 (14.0)	53.93 (13.0)	52.70 (12.9)	<0.001
Social	65.41 (21.7)	64.69 (21.3)	63.40 (20.8)	0.001
Environmental	52.80 (16.3)	52.46 (15.5)	51.36 (15.8)	<0.001
**QOL by Setting**	**Rural Mean (SD)**	**Urban Mean (SD)**	**-**	**P-value**
Physical	54.18 (11.4)	55.95 (11.0)	-	<0.001
Psychological	53.55 (13.3)	55.00 (12.4)	-	<0.001
Social	64.04 (21.2)	68.10 (21.2)	-	<0.001
Environmental	51.88 (15.7)	55.19 (15.3)	-	<0.001

Case wise total, valid & missing cases are given in Appendix Table A in S1 Text.

For the physical domain, mean QOL scores were 54.9 (SD = 10.7) for males and 53.8 (SD = 11.9) for females (P < 0.001). Similar trends were observed in the psychological, social, and environmental domains, with higher QOL scores in males.

In the physical domain, mean scores were 54.6 (SD = 11.8), 54.6 (SD = 11.2), and 53.6 (SD = 11.3) for the three age groups: 18–29, 30–49, and ≥50, respectively (P = 0.001). Significant differences were observed among the age groups (P = 0.001), indicating that older individuals reported lower QOL scores in the physical domain. Similar patterns were observed in the psychological, social, and environmental domains, with the lowest QOL scores in the older age groups.

For rural and urban settings, significant differences were observed across all domains. For the physical domain, mean QOL scores were 54.2 (SD = 11.4) for rural and 55.9 (SD = 11.0) for urban (P < 0.001). Similar patterns were noted in the psychological, social, and environmental domains, with lower QOL scores in rural settings.

### Non-communicable diseases (NCDs)

Table 5 summarises the findings on the prevalence of NCDs in Khyber Pakhtunkhwa, Pakistan. Hypertension stood out with the highest prevalence rate at 29.2%, followed by renal diseases (7.6%) and diabetes (6.2%). Other NCDs, including hypercholesterolemia, asthma, epilepsy, coronary heart disease, cancer, and thalassemia, exhibited lower overall prevalence rates ranging from 0.7% to 3.0%.

**Table 5:** Non communicable diseases (NCDs), communicable diseases (CDs), and disabilities stratified by sex, age, and setting.

Disease	All Cases	Sex			Age Group				Setting		
		Male	Females	P-value	18–29	30–49	≥50	P-value	Rural	Urban	P-value
**Non-communicable diseases (NCDs)**											
Hypertension	4242 (29.2%)	1703 (25.0%)	2516(32.7%)	< .001	361 (15.3%)	2300 (28.1%)	1573 (40.4%)	< .001	3764 (29.1%)	478 (29.6%)	0.66
Hypercholesterolemia	407 (3.0%)	125 (1.9%)	280 (3.9%)	< .001	55 (2.4%)	256(3.4%)	96 (2.7%)	0.26	366 (3.0%)	41 (2.8%)	0.71
Diabetes	852 (6.2%)	481 (7.4%)	367 (5.2%)	< .001	58 (2.5%)	383 (5.0%)	407 (11.2%)	< .001	720 (5.9%)	132 (9.0%)	<0.001
Renal diseases	1034 (7.6%)	426 (6.6%)	599(8.3%)	< .001	131 (5.7%)	662 (8.1%)	277 (7.7%)	< .001	961 (7.9%)	73 (5.0%)	<0.001
Asthma	347 (2.6%)	164 (2.6%)	181(2.6%)	0.72	47 (2.1%)	184 (2.4%)	116 (3.2%)	< .001	312 (2.6%)	35 (2.5%)	0.007
Epilepsy	112 (0.8%)	61 (1.0%)	50 (0.7%)	0.05	10 (0.5%)	61 (0.8%)	40 (1.1%)	0.09	106 (0.9%)	6 (0.5%)	0.01
Coronary Heart Disease	396 (2.9%)	183 (2.8%)	209 (3.0%)	0.65	35 (1.5%)	200 (2.6%)	161 (4.5%)	< .001	351 (2.9%)	45 (3.1%)	0.66
Cancer	100 (0.7%)	51 (0.7%)	48 (0.7%)	0.82	17 (0.7%)	41 (0.5%)	12 (0.3%)	0.16	90 (0.7%)	10 (0.5%)	0.09
Thalassemia	60 (0.9%)	31 (1.0%)	29 (0.8%)	0.47	12 (1.1%)	35 (1.0%)	14 (0.8%)	0.02	52 (0.9%)	8 (1.3%)	<0.001
**Communicable diseases (CDs)**											
Hepatitis B	72 (0.5%)	16 (0.3%)	55 (0.8%)	<0.001	7 (0.4%)	43 (0.6%)	22 (0.6%)	0.24	69 (0.6%)	3 (0.2%)	0.07
Hepatitis C	110 (0.8%)	44 (0.7%)	63 (0.9%)	0.163	10 (0.5%)	74 (1.0%)	25 (0.7%)	0.29	102 (0.8%)	8 (0.6%)	0.25
Tuberculosis	69 (0.5%)	29 (0.5%)	38 (0.5%)	0.45	0 (0.0%)	41 (0.5%)	28 (0.8%)	<0.001	64 (0.5%)	5 (0.4%)	0.362
AIDS	45 (0.3%)	0 (0.0%)	45 (0.6%)	<0.001	7 (0.3%)	24 (0.3%)	14 (0.4%)	0.785	39 (0.3%)	6 (0.4%)	0.56
**Disabilities**											
Congenital	601 (3.2%)	82 (0.9%)	515 (5.3%)	<0.001	102 (3.2%)	324 (3.0%)	174 (3.4%)	0.496	587 (3.5%)	14 (0.6%)	0.03
Post-injury	383 (2.0%)	195 (2.1%)	179 (1.8%)	<0.001	62 (2.0%)	185 (1.7%)	127 (2.5%)	<0.001	323 (1.9%)	60 (2.4%)	0.001
Post-disease	242 (1.3%)	139 (1.5%)	102 (1.0%)	<0.001	17 (0.5%)	138 (1.3%)	86 (1.7%)	<0.001	212 (1.3%)	30 (1.2%)	0.05
Paralysis	282 (2.1%)	135 (2.1%)	146 (2.1%)	0.835	41 (1.8%)	126 (1.7%)	115 (3.3%)	<0.001	256 (2.1%)	26 (1.8%)	0.435

Case wise total, valid & missing cases are given in Appendix Table A in S1 Text.

Hypertension, hypercholesterolemia, and renal diseases showed higher prevalence rates in females (32.7%, 3.9%, and 8.3%, respectively) compared to males (25.0%, 1.9%, and 6.6%, respectively, P < 0.001). In contrast, diabetes was highly prevalent among males (7.4%) compared to females (5.2%, P < 0.001). Conditions such as asthma, coronary heart disease, and cancer did not exhibit statistically significant differences between the sexes (P = 0.72, P = 0.65, and P = 0.82, respectively).

There was a significant variation in NCD prevalence across different age groups. Hypertension and coronary heart diseases exhibited a significant increase with age (P < 0.001), with the highest prevalence in participants aged 50 and above (40.4% and 4.5%, respectively). Similarly, diabetes prevalence showed a significant rise with age, reaching its peak in the ≥50 age group (11.2%) compared to 2.5% in the 18–29 years’ age group and 5.0% in the 30–49 years’ age group (P < 0.001).

Diabetes and thalassemia showed slightly higher prevalence rates in urban settings (9.0% and 1.3%, respectively) than in rural settings (5.9% and 0.9%, respectively) (P < 0.001). Conversely, there was no statistically significant difference in the prevalence of hypertension, hypercholesterolemia, and coronary heart diseases between rural and urban areas (P = 0.6, P = 0.7, and P = 0.6, respectively).

### Communicable diseases (CDs)

Burden of CDs are presented in Table 5. Hepatitis C showed an overall prevalence rate of 0.8%, followed by TB (0.5%), hepatitis B (0.5%), and AIDS (0.3%).

Significant differences were observed between males and females for hepatitis B (P < 0.001), with females having a higher prevalence (0.8%) compared to males (0.3%). Similarly, AIDS also demonstrated a significant sex-based difference (P < 0.001), with females having a higher prevalence (0.6%) compared to males (0%).

Hepatitis B and C showed no significant variation with age (P = 0.24, P = 0.29), with the lowest prevalence in the 18–29 age group (0.4%, 0.5% respectively). TB demonstrated a significant increase in prevalence with age (P < 0.001), reaching the highest level in the ≥50 years’ age group (0.8%).

Hepatitis B demonstrated higher prevalence rates in rural areas (0.6%) than in urban areas (0.2%, P = 0.07). Similarly, hepatitis C and TB did not show statistically significant differences in prevalence between urban and rural settings (P = 0.25 and P = 0.56, respectively).

### Disabilities

Results for the prevalence of disabilities are presented in Table 5. Congenital disabilities revealed a statistically significant gender disparity, with a prevalence of 5.3% in females compared to 0.9% in males (P < 0.001). Similarly, post-injury disabilities showed a significant difference, with 2.1% of males and 1.8% of females having such conditions (P < 0.001). Post-disease disabilities also demonstrated significant gender-based variations, with a prevalence of 1.5% in males and 1.3% in females (P < 0.001). Regarding paralysis disabilities, the prevalence was 2.1% in both males and females, with no significant difference (P = 0.835).

Congenital disabilities showcased an overall prevalence of 3.2%, with no significant variations across age groups (3.2% in 18–29, 3.0% in 30–49, and 3.4% in ≥50, P = 0.496). In contrast, post-injury and post-disease disabilities exhibited significant age-related differences (P < 0.001). For post-injury disabilities, the prevalence was 2.0% overall, with the lowest (1.7%) in the 30–49 age group. Post-disease disabilities also displayed significant age-related variations, with a prevalence of 1.3% overall, increasing from 0.5% in the 18–29 age group to 1.3% in the 30–49 age group and further to 1.7% in the ≥50 age group. Similarly, paralysis demonstrated significant age-related trends, with the highest prevalence in the ≥50 age group (3.3%) compared to the 18–29 (1.8%) and 30–49 (1.7%) age groups (P < 0.001).

The prevalence of congenital disabilities was 3.5% in rural settings and 0.6% in urban settings, revealing a statistically significant difference (P = 0.03). For post-injury disabilities, the prevalence was 1.9% in rural settings and 2.4% in urban settings, showing a significant difference (P = 0.001). Post-disease disabilities also displayed a significant difference, with a prevalence of 1.3% in rural areas and 1.2% in urban areas (P = 0.05). However, for paralysis, there was no significant difference (P = 0.435) in rural (2.1%) and urban (1.8%) settings.

## Discussion

This study provides a comprehensive evaluation of the CDs, NCDs, disabilities and related risk factors across all 7 divisions in Khyber Pakhtunkhwa, Pakistan. Though past research has explored the patterns of CDs, NCDs and disabilities in Pakistan and Khyber Pakhtunkhwa, the evidence on the distribution of these health conditions across sex, age categories and settings is scarce.

Our findings indicate that females are predominantly in the pre-obesity or obesity category and are engaged in low levels of physical activity. They also exhibit a high prevalence of certain NCDs and CDs included in our study and tend to have comparatively lower quality of life. These findings are consistent with previous research conducted in LMICs, including those in South Asia, and with data from the World Health Organisation [55–57]. The results of our study indicate more than half (52.0%) of the female participants were pre-obese or obese, which is consistent with the results of the Pakistan Demographic and Health Survey 2017–18, where 52% of females in Khyber Pakhtunkhwa were found to be pre-obese or obese [58]. These results indicate this segment of the population is at high risk for developing NCDs like type 2 diabetes and hypertension; 90% of people with type 2 diabetes [59] and 75% of people with hypertension are either overweight or obese [60].

Among NCDs, hypertension prevalence was the highest, with a high female-to-male ratio, a finding corroborated by previous literature that also identified hypertension as the most common NCD in Pakistan [61, 62]. The prevalence of most NCDs is higher in the elderly population (≥50 age). A similar trend of NCD prevalence was reported in a study by the World Health Organisation on global ageing and adult health from six countries across the globe, which likewise indicated that the prevalence of NCDs increases with age [63]. Similar findings indicating age and sex disparities in the burden of NCDs have been evident in other LMICs, including those in South Asia [64–66]. Our findings show a significant prevalence of renal diseases in rural settings, aligning with existing literature in a similar context. This may be due to a combination of factors, including limited access to healthcare resources, inadequate disease surveillance, complex sociodemographic transitions, rapid urbanisation, and changes in diet in rural areas [67–71]. In contrast, certain NCDs, such as diabetes and thalassemia were more prevalent in the urban settings, consistent with previous literature in a comparable context [72–74]. The lower prevalence of certain non-communicable diseases (NCDs), such as diabetes (6%), observed in our survey compared to other similar surveys like the Diabetes Prevalence Survey of Pakistan (DPS-PAK) [75], can be attributed to the self-report nature of our survey. Self-reported surveys often miss a high proportion of undiagnosed cases. Additionally, our survey was conducted in 2016–2017. Given the rising rates of NCDs, particularly diabetes, the self-reported prevalence from our survey, if extrapolated to the present, would likely yield prevalence rates comparable to those reported in other surveys where objective methods were used to measure disease burden. Addressing this high burden of NCD requires urgent action, including increased screening, prevention, detection, and treatment efforts at primary healthcare levels throughout the province especially rural settings where over 70% of the population resides.

In line with existing evidence, among the CDs, hepatitis B and Hepatitis C are more prevalent than HIV(AIDS) [14, 15, 76]. Hepatitis B was more prevalent in females than males. This finding is consistent with previous literature from Pakistan, where 52.24% of the hepatitis B patients were females [77–79]. However, our results deviate from global trends in the context of AIDS, as the global trends show that generally young men are likely to be diagnosed with AIDS than women [80, 81]. In contrast, our study revealed that AIDs was not reported by any male. This discrepancy can be attributed to the concept of harmful masculinity and male stereotypes, which create situations that make having safer sex, testing for HIV, accessing and complying with treatment, and even discussing sexuality difficult for men. Consequently, men may choose not to self-report their condition. This is a major concern because it not only affects male health outcomes but also contributes to the spread of HIV, which affects women and girls as well [82–84]. Addressing these disparities requires gender-sensitive strategies and person-centred services to effectively reach and engage men in HIV prevention, testing, and treatment programmes [85–87].

Disabilities such as post-disease disabilities and paralysis tend to increase with age. Similar results have been reported by the Research Consortium on Educational Outcomes and Poverty (RECOUP) working group indicating that disabilities increase gradually by age [32]. Congenital disabilities were the most frequent of all the disabilities, as well as higher in female participants; while post-injury and post-disease were higher in men. Findings from existing literature also indicate that men are more likely than women to experience work-related disabilities [88, 89], On the contrary, males have previously been reported as the disadvantaged sex for congenital disabilities compared to females [90]. Our findings may be attributed to disparities in prenatal care access and quality, as inadequate healthcare during pregnancy could contribute to the development of congenital anomalies [91]. Maternal nutrition represents another significant factor, where variations in dietary practices have the potential to influence foetal development [92]. Another potential factor might be that men tend to underreport or overlook their condition due to societal or cultural influences [93, 94]. Further investigation is essential to examine potential regional, genetic, or environmental factors contributing to this variation.

The present study has several advantages over earlier ones. We determined CDs, NCDs, and disabilities stratified by sex, age and setting, comprehensively analysing their distribution and impact. The findings of our study are generalisable to similar populations of the lower-middle-income countries with comparable demographic characteristics, cultural norms and healthcare systems. Given the triple burden of CDs, NCDs, and disabilities, as well as related risk factors, an organised approach is required to strengthen health policy and legislative frameworks. Improving primary health care and ensuring universal health coverage are essential steps towards meeting Sustainable Development Goal 3. Pakistan must rapidly adopt and implement the guidance outlined in the National Health Vision Pakistan 2016–2025 to achieve these objectives [95] and improve the country’s access, coverage, and quality of disease prevention and control interventions. Additionally, integrated NCD service delivery at the primary healthcare level, supported by evidence-based guidelines, essential technologies, a well-trained health workforce, and robust health information systems, is also required to control the disease burden effectively. Incorporating and prioritising the WHO’s best buys in NCD prevention [96] can further enhance the effectiveness of these interventions and contribute to the overall health and well-being of the population, thus achieving SDG targets.

### Limitations

It is important to acknowledge certain limitations in our study. A more accurate estimation of burdens is crucial for effective policymaking and monitoring of public health interventions. By analysing risk factors associated with NCDs and CDs and conducting a time series analysis of burden trends, we can enhance our understanding and inform policy decisions. The diseases were self-reported based on participants’ previous medical history, without validation through laboratory tests or examinations during data collection. This approach may have led to underestimation, particularly for undiagnosed cases of both CDs and NCDs. Furthermore, missing data posed another significant limitation. While we used pairwise deletion, which may affect the robustness of our findings, it’s worth noting that this method, under Missing Completely at Random (MCAR) or Missing at Random (MAR) assumptions, tends to be less biased and better preserves information compared to other deletion methods [54].

## Conclusions

Our research supports the need for comprehensive prevention strategies to address CDs, NCDs, and disabilities in Pakistan. While efforts are being made to achieve universal health coverage in the country, most interventions thus far have focused on the curative aspect of healthcare. Given the context of a developing nation like Pakistan, prioritising preventive interventions is paramount to effectively mitigating the heightened risk associated with CDs, NCDs, and disabilities. To tackle these challenges, formulating and implementing a coherent national health policy that emphasises enhancing public health capacity and establishing dedicated wings for CDs, NCDs, and disabilities within the provinces is essential. Furthermore, improved governance and resource allocation, coupled with active engagement from both public and private sectors, are imperative.

Establishing inter-sectoral collaborations with pertinent organisations to formulate key policy interventions and preventive measures is crucial. By adopting these measures, Pakistan can substantially alleviate the disease burden and enhance the overall health and well-being of its population.

## Supporting information

S1 Checklist STROBE statement—checklist of items that should be included in reports of observational studies. (DOCX)

S1 Text Appendix Table A. Case wise total, valid & missing data across variables.
(DOCX)
