# Supplementary material for: Prevalence of communicable, non-communicable diseases, disabilities and related risk factors in Khyber Pakhtunkhwa Pakistan: Findings from the Khyber Pakhtunkhwa Integrated Population and Health Survey (2016–17)
Source: PLoS One. 2025 Feb 3;20(2):e0308209. doi: 10.1371/journal.pone.0308209 (PMC11790158; doi:10.1371/journal.pone.0308209)
Supplement: S1 Checklist — (DOCX) [file pone.0308209.s001.docx]

STROBE Statement—checklist of items that should be included in reports of observational studies

|  | Item No. | Recommendation | Page  No. | Relevant text from manuscript |
| --- | --- | --- | --- | --- |
| **Title and abstract** | 1 | (a) Indicate the study’s design with a commonly used term in the title or the abstract | 2 | This cross-sectional survey was conducted in 24 districts across all 7 divisions of Khyber Pakhtunkhwa on population ageing ≥18 years. |
|  |  | (b) Provide in the abstract an informative and balanced summary of what was done and what was found | 2 | Mentioned under the Methodology and Results subheadings in the Abstract section. |
| Introduction | | | |  |
| Background/rationale | 2 | Explain the scientific background and rationale for the investigation being reported | 3,4 | Mentioned for all the three domains of interest: CDs, NCDs and disabilities in the Introduction section. |
| Objectives | 3 | State specific objectives, including any prespecified hypotheses | 3,4 | We conducted the Khyber Pakhtunkhwa Integrated Population and Health Survey (KP-IPHS), which aims to fill these gaps by providing comprehensive data on various health indicators, including the leading CDs, NCDs, and disabilities in the province; and to influence local health policy frameworks in the country. It also aims to provide valuable information for the global dialogue on healthcare disparities in Pakistan and to guide international health agendas by advocating for strategies that promote universal health coverage and equitable healthcare access and delivery in LMICs including Pakistan. |
| Methods | | | |  |
| Study design | 4 | Present key elements of study design early in the paper | 4,5 | This cross-sectional survey was conducted in 24 districts of Khyber Pakhtunkhwa, ensuring a balanced representation of all 7 divisions of Khyber Pakhtunkhwa and the diverse geographical and demographic characteristics of the province. |
| Setting | 5 | Describe the setting, locations, and relevant dates, including periods of recruitment, exposure, follow-up, and data collection | 5,6 | Mentioned under the “Materials and methods” and “Sample size and sampling strategy” subheadings. |
| Participants | 6 | (a) Cohort study—Give the eligibility criteria, and the sources and methods of selection of participants. Describe methods of follow-up  Case-control study—Give the eligibility criteria, and the sources and methods of case ascertainment and control selection. Give the rationale for the choice of cases and controls.  Cross-sectional study—Give the eligibility criteria, and the sources and methods of selection of participants | 5,6 | Mentioned under the “Eligibility criteria” and “Sample size and sampling strategy” subheadings. |
|  |  | (b) Cohort study—For matched studies, give matching criteria and number of exposed and unexposed  Case-control study—For matched studies, give matching criteria and the number of controls per case | Not Applicable | Not Applicable |
| Variables | 7 | Clearly define all outcomes, exposures, predictors, potential confounders, and effect modifiers. Give diagnostic criteria, if applicable | 5,6,7,8 | Outcomes and predictors are given in the Methodology section. |
| Data sources/ measurement | 8* | For each variable of interest, give sources of data and details of methods of assessment (measurement). Describe comparability of assessment methods if there is more than one group | 5,6,7,8 | Given for each variable/domain of interest |
| Bias | 9 | Describe any efforts to address potential sources of bias | 8 | To deal with the missing data, we employed pairwise deletion, a method that is well-suited to Missing Completely at Random (MCAR) or Missing at Random (MAR) data.[54] Given the variation in missing data across variables in our study, pairwise deletion was deemed advantageous as it is less biased under MCAR or MAR assumptions and preserves more information than other deletion methods. |
| Study size | 10 | Explain how the study size was arrived at | 6 | Mentioned under the “Sample size and sampling strategy” subheadings. |

Continued on next page

| Quantitative variables | 11 | Explain how quantitative variables were handled in the analyses. If applicable, describe which groupings were chosen and why | 5 to 8 | Given for each variable and subgroups. |
| --- | --- | --- | --- | --- |
| Statistical methods | 12 | (a) Describe all statistical methods, including those used to control for confounding | 7,8 | Given under the subheading “Data Handling and Analysis“ |
|  |  | (b) Describe any methods used to examine subgroups and interactions | 8 | Descriptive statistics, including frequency percentages for categorical variables, and means with standard deviation (SD) for scale variables, are presented. The independent t-test and One-way ANOVA were used to compare means between two and more than two independent variables, respectively. Stratification of the prevalence of CDs, NCDs and disabilities across various demographic characteristics such as sex, age, and setting was conducted. Pearson’s Chi-squared test was employed to evaluate significant differences in prevalence between groups. A P-value of 0.05 or less was considered statistically significant, whereas a P-value of 0.05 or more was considered to indicate no association between these. For reporting all results from the survey data, sample weights are used. |
|  |  | (c) Explain how missing data were addressed | 8 | To deal with the missing data, we employed pairwise deletion, a method that is well-suited to Missing Completely at Random (MCAR) or Missing at Random (MAR) data.[54] Given the variation in missing data across variables in our study, pairwise deletion was deemed advantageous as it is less biased under MCAR or MAR assumptions and preserves more information than other deletion methods. |
|  |  | (d) Cohort study—If applicable, explain how loss to follow-up was addressed  Case-control study—If applicable, explain how matching of cases and controls was addressed  Cross-sectional study—If applicable, describe analytical methods taking account of sampling strategy | 5 | The survey employed a multistage stratified cluster sampling technique. |
|  |  | (e) Describe any sensitivity analyses | N/A | N/A |
| Results | | | | |
| Participants | 13* | (a) Report numbers of individuals at each stage of study—eg numbers potentially eligible, examined for eligibility, confirmed eligible, included in the study, completing follow-up, and analysed | N/A | N/A (cross-sectional study) |
|  |  | (b) Give reasons for non-participation at each stage | N/A | N/A |
|  |  | (c) Consider use of a flow diagram | N/A | N/A |
| Descriptive data | 14* | (a) Give characteristics of study participants (eg demographic, clinical, social) and information on exposures and potential confounders | 9,10 | Mentioned under the subheading “Demographic characteristics of study participants” in the Results Section. |
|  |  | (b) Indicate number of participants with missing data for each variable of interest | 8 to 17 | The tables contain the total response data for each variable. Details of missing data is given in appendix A. For 88% of variables less than 30% of data is missing. Pair wise deletion was used and justification has been provided in the manuscript. |
|  |  | (c) Cohort study—Summarise follow-up time (eg, average and total amount) | N/A | N/A |
| Outcome data | 15* | Cohort study—Report numbers of outcome events or summary measures over time | N/A | N/A |
|  |  | Case-control study—Report numbers in each exposure category, or summary measures of exposure | N/A | N/A |
|  |  | Cross-sectional study—Report numbers of outcome events or summary measures | 8 to 17 | Mentioned for each domain of interest separately under subheadings in the Results section. |
| Main results | 16 | (a) Give unadjusted estimates and, if applicable, confounder-adjusted estimates and their precision (eg, 95% confidence interval). Make clear which confounders were adjusted for and why they were included | 8 to 17 | Point and interval estimates have been provided. Significance level is set at 0.05 in the tables where needed. |
|  |  | (b) Report category boundaries when continuous variables were categorized | 8 to 17 | Mentioned for age |
|  |  | (c) If relevant, consider translating estimates of relative risk into absolute risk for a meaningful time period | N/A | N/A |

Continued on next page

| Other analyses | 17 | Report other analyses done—e.g., analyses of subgroups and interactions, and sensitivity analyses | 8 to 17 | Sub-groups analysis is given in the tables |
| --- | --- | --- | --- | --- |
| Discussion | | | | |
| Key results | 18 | Summarise key results with reference to study objectives | 17,18,19 | Mentioned in the discussion section for each domain: CDs, NCDs and disabilities. |
| Limitations | 19 | Discuss limitations of the study, taking into account sources of potential bias or imprecision. Discuss both direction and magnitude of any potential bias | 19 | Mentioned under the subheading “Limitations” in the Discussion section. |
| Interpretation | 20 | Give a cautious overall interpretation of results considering objectives, limitations, multiplicity of analyses, results from similar studies, and other relevant evidence | 17,18,19 | Given in the discussion section for each domain: CDs, NCDs and disabilities. |
| Generalisability | 21 | Discuss the generalisability (external validity) of the study results | 18 | The findings of our study are generalisable to similar populations of the lower-middle-income countries with comparable demographic characteristics, cultural norms and healthcare systems. |
| Other information | |  | | |
| Funding | 22 | Give the source of funding and the role of the funders for the present study and, if applicable, for the original study on which the present article is based | 17 | The Population Welfare Department of Khyber Pakhtunkhwa conducted this study with the United Nations Population Fund (UNFPA) financial support. The Institute of Public Health KMU provided technical assistance, including questionnaire design, sample size calculation and sample selection (with the assistance of PBS), data management, data analysis, and report writing. |

*Give information separately for cases and controls in case-control studies and, if applicable, for exposed and unexposed groups in cohort and cross-sectional studies.

**Note:** An Explanation and Elaboration article discusses each checklist item and gives methodological background and published examples of transparent reporting. The STROBE checklist is best used in conjunction with this article (freely available on the Web sites of PLoS Medicine at http://www.plosmedicine.org/, Annals of Internal Medicine at http://www.annals.org/, and Epidemiology at http://www.epidem.com/). Information on the STROBE Initiative is available at www.strobe-statement.org.
