# Supplementary material for: Prevalence of communicable, non-communicable diseases, disabilities and related risk factors in Khyber Pakhtunkhwa Pakistan: Findings from the Khyber Pakhtunkhwa Integrated Population and Health Survey (2016–17)
Source: PLoS One. 2025 Feb 3;20(2):e0308209. doi: 10.1371/journal.pone.0308209 (PMC11790158; doi:10.1371/journal.pone.0308209)
Supplement: S1 Text — Appendix Table A. Case wise total, valid & missing data across variables. (DOCX) [file pone.0308209.s002.docx]

**Appendix Table A. Case wise total, valid & missing data across variables.**

| Parameter | Missing Cases | Valid Cases | Total Participants | Missing Data % |
| --- | --- | --- | --- | --- |
| Sex | 116 | 18951 | 19067 | 0.60% |
| Age Categories | 151 | 18916 | 19067 | 0.80% |
| Education | 11384 | 7683 | 19067 | 59.70% |
| Setting | 0 | 20704 | 19067 | 0% |
| BMI | 6249 | 12818 | 19067 | 32.80% |
| BMI *Sex | 6281 | 12786 | 19067 | 32.90% |
| BMI *Age | 6351 | 12716 | 19067 | 33.30% |
| BMI *Setting | 6250 | 12817 | 19067 | 32.80% |
| Hypertension | 4520 | 14547 | 19067 | 23.70% |
| Hypertension*Sex | 4558 | 14509 | 19067 | 23.9%% |
| Hypertension*Age | 4622 | 14445 | 19067 | 24.2%% |
| Hypertension*Setting | 4519 | 14548 | 19067 | 23.7%% |
| Hypercholesterolemia | 5503 | 13564 | 19067 | 28.90% |
| Hypercholesterolemia*Sex | 5523 | 13544 | 19067 | 29.00% |
| Hypercholesterolemia*Age | 5604 | 13463 | 19067 | 29.40% |
| Hypercholesterolemia*Setting | 5502 | 13565 | 19067 | 28.90% |
| Diabetes | 5441 | 13626 | 19067 | 28.50% |
| Diabetes*Sex | 5463 | 13604 | 19067 | 28.70% |
| Diabetes*Age | 5543 | 13524 | 19067 | 29.10% |
| Diabetes*Setting | 5440 | 13627 | 19067 | 28.50% |
| Renal | 5393 | 13674 | 19067 | 28.30% |
| Renal*Sex | 5419 | 13648 | 19067 | 28.40% |
| Renal*Age | 5496 | 13571 | 19067 | 28.80% |
| Renal*Setting | 5392 | 13675 | 19067 | 28.30% |
| Asthma | 5595 | 13472 | 19067 | 29.30% |
| Asthma*Sex | 5615 | 13452 | 19067 | 29.40% |
| Asthma*Age | 5696 | 13371 | 19067 | 29.90% |
| Asthma*Setting | 5595 | 13472 | 19067 | 29.30% |
| Epilepsy | 5637 | 13430 | 19067 | 29.60% |
| Epilepsy*Sex | 5657 | 13410 | 19067 | 29.70% |
| Epilepsy*Age | 5741 | 13326 | 19067 | 30.10% |
| Epilepsy*Setting | 5637 | 13430 | 19067 | 29.60% |
| Coronary Heart Disease | 5574 | 13493 | 19067 | 29.20% |
| Coronary Heart Disease*Sex | 5596 | 13471 | 19067 | 29.30% |
| Coronary Heart Disease*Age | 5648 | 13419 | 19067 | 29.60% |
| Coronary Heart Disease*Setting | 5574 | 13493 | 19067 | 29.20% |
| Cancer | 4428 | 14639 | 19067 | 23.20% |
| Cancer*Sex | 4473 | 14594 | 19067 | 23.50% |
| Cancer*Age | 4549 | 14518 | 19067 | 23.90% |
| Cancer*Setting | 4428 | 14639 | 19067 | 23.20% |
| Thalassemia | 12547 | 6520 | 19067 | 65.80% |
| Thalassemia*Sex | 12576 | 6491 | 19067 | 66.00% |
| Thalassemia*Age | 12616 | 6451 | 19067 | 66.20% |
| Thalassemia*Setting | 12548 | 6519 | 19067 | 65.80% |
| Hepatitis B | 5620 | 13447 | 19067 | 29.50% |
| Hepatitis B*Sex | 5640 | 13427 | 19067 | 29.60% |
| Hepatitis B*Age | 5724 | 13343 | 19067 | 30.00% |
| Hepatitis B*Setting | 5620 | 13447 | 19067 | 29.50% |
| Hepatitis C | 5606 | 13461 | 19067 | 29.40% |
| Hepatitis C*Sex | 5627 | 13440 | 19067 | 29.50% |
| Hepatitis C*Age | 5708 | 13359 | 19067 | 29.90% |
| Hepatitis C*Setting | 5606 | 13461 | 19067 | 29.40% |
| Tuberculosis | 5613 | 13454 | 19067 | 29.40% |
| Tuberculosis*Sex | 5634 | 13433 | 19067 | 29.50% |
| Tuberculosis*Age | 5715 | 13352 | 19067 | 30.00% |
| Tuberculosis*Setting | 5614 | 13453 | 19067 | 29.40% |
| AIDS | 5622 | 13445 | 19067 | 29.50% |
| AIDS*Sex | 5640 | 13427 | 19067 | 29.60% |
| AIDS*Age | 5696 | 13371 | 19067 | 29.90% |
| AIDS*Setting | 5622 | 13445 | 19067 | 29.50% |
| Disability | 4717 | 14350 | 19067 | 24.70% |
| Disability*Sex | 4767 | 14300 | 19067 | 25.00% |
| Disability*Age | 4837 | 14230 | 19067 | 25.40% |
| Disability*Setting | 4717 | 14350 | 19067 | 24.70% |
| METs | 8246 | 10821 | 19067 | 56.80% |
| METs *Sex | 8231 | 10836 | 19067 | 56.80% |
| METs *Age | 8173 | 10894 | 19067 | 57.10% |
| METs *Setting | 8246 | 10821 | 19067 | 56.80% |
| Congenital Disability | 4717 | 14350 | 19067 | 24.70% |
| Congenital Disability*Sex | 4767 | 14300 | 19067 | 25.00% |
| Congenital Disability*Age | 4837 | 14230 | 19067 | 25.40% |
| Congenital Disability*Setting | 4717 | 14350 | 19067 | 24.70% |
| Post-injury Disability | 4717 | 14350 | 19067 | 24.70% |
| Post-injury Disability*Sex | 4767 | 14300 | 19067 | 25.00% |
| Post-injury Disability*Age | 4837 | 14230 | 19067 | 25.40% |
| Post-injury Disability*Setting | 4717 | 14350 | 19067 | 24.70% |
| Post-disease Disability | 4717 | 14350 | 19067 | 24.70% |
| Post-disease Disability*Sex | 4767 | 14300 | 19067 | 25.00% |
| Post-disease Disability*Age | 4837 | 14230 | 19067 | 25.40% |
| Post-disease Disability*Setting | 4717 | 14350 | 19067 | 24.70% |
| Paralysis | 5678 | 13389 | 19067 | 29.80% |
| Paralysis *Sex | 5698 | 13369 | 19067 | 29.90% |
| Paralysis *Age | 5780 | 13287 | 19067 | 30.30% |
| Paralysis *Setting | 5679 | 13388 | 19067 | 29.80% |
